# Music‐based interventions for nonfluent aphasia: A systematic review of randomized control trials

**DOI:** 10.1111/nyas.15387

**Published:** 2025-06-21

**Authors:** Yuko Koshimori, Preetie Shetty Akkunje, Evelyn Tjiandri, Julia B. Kowaleski, Michael H. Thaut

**Affiliations:** ^1^ Music and Health Science Research Collaboratory, Faculty of Music University of Toronto Toronto Ontario Canada; ^2^ Department of Audiology and Speech Language Pathology Kasturba Medical College Mangalore, Manipal Academy of Higher Education Manipal India; ^3^ Institute of Medical Science, Temerty Faculty of Medicine University of Toronto Toronto Ontario Canada

**Keywords:** music‐based interventions, nonfluent aphasia recovery, quality assessment, randomized controlled trials, risk of bias, systematic review

## Abstract

Music‐based interventions (MBIs) offer promising strategies for addressing speech‐language impairments in individuals with nonfluent aphasia. This systematic review summarizes the current literature of MBIs for nonfluent aphasia recovery by types of MBIs to determine the efficacy of MBIs and assesses the risk of bias to identify common methodological limitations. A systematic search was conducted of MEDLINE, PubMed, and APA PsycInfo for the 20 years preceding July 2024. Risk of bias assessment was performed using the revised Joanna Briggs Institute critical appraisal tool for randomized controlled trials (RCTs). Ten RCTs met the inclusion criteria, featuring MBIs such as Melodic Intonation Therapy, Modified Melodic Intonation Therapy, and singing‐based approaches. The results highlighted the potential of MBIs in various domains, particularly in enhancing repetition and naming abilities, even when compared to speech therapy. The reviewed studies exhibited a moderate to high risk of bias. Outcome measures varied widely, and functional communication, a critical rehabilitation goal, was examined in just two RCTs. Furthermore, heterogeneous control conditions and statistical methods hindered meaningful comparisons across studies. Future research should prioritize functional communication outcomes and refine intervention protocols to strengthen the evidence base. Addressing these gaps is essential for advancing the potential benefits of these clinical tools for nonfluent aphasia recovery.

## Graphical abstract

This systematic review analyzes 10 randomized control trials of music‐based interventions (MBIs) for nonfluent aphasia, including Melodic Intonation Therapy and singing‐based approaches. Results showed MBIs could enhance repetition and naming abilities—highlighting their therapeutic potential. However, methodological inconsistencies and biases limit the strength of evidence. Standardized methodologies, uniform metrics, and rigorous statistical frameworks are needed to enhance the robustness of evidence and optimize MBI applications in clinical practice.

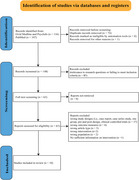

## INTRODUCTION

Nonfluent aphasia, a language disorder commonly resulting from stroke or traumatic brain injury, is characterized by impaired speech production, reduced fluency, and difficulty in language comprehension. Beyond traditional interventional methods, researchers have explored the potential of music‐based interventions (MBIs) to address these deficits, leveraging the preserved musical and rhythmic abilities often observed in individuals with aphasia.^1^ Music in rehabilitation has been widely studied across various neurological disorders, including Parkinson's disease (PD) and dementia. For nonfluent aphasia, growing evidence supports the use of music‐based approaches to facilitate language recovery and improve communication.

The shared neuroanatomical and functional mechanisms between music and speech have spurred interest in MBIs as tools to promote language recovery through neuroplasticity.^2^ Singing, for example, has shown to activate language areas in the brain that may have been spared from the initial injury, allowing patients to rewire their language centers and recover their ability to speak.^3^, ^4^ This phenomenon is supported by neuroimaging studies that have reported the recruitment of sensorimotor areas during music listening, which enhances motor connections in stroke patients undergoing musical intervention,^3^, ^4^, ^5^ indicating that these innovative strategies offer promising solutions to the complex communication challenges associated with nonfluent aphasia.

Both music and speech are structured auditory communication systems characterized by acoustic parameters, hierarchical syntactic structure, and rhythm.^6^, ^7^, ^8^ They engage overlapping neural networks for perception and production.^9^, ^10^ Music and language also share similar rhythmic structures, organizing sounds into phrases marked by pauses and changes in tone. In both, these phrases create a sense of timing and flow. For example, English has a rhythmic pattern where stressed syllables occur at regular intervals, known as isochronous stress rhythm. This shared rhythm explains why techniques from music can improve speech fluency and timing in language therapy.^7^, ^11^ The rhythmic congruence between music and speech enables auditory rhythm to enhance speech fluency, articulation, pause timing, intelligibility, and well‐timed and precise controlled muscle movements required for speech production.^12^, ^13^

Research has indicated that MBIs such as Melodic Intonation Therapy (MIT), Modified Melodic Intonation Therapy (MMIT), choral singing, singing therapy, or song writing engage undamaged brain regions to facilitate the recovery of speech (comprehension and production) in individuals with stroke, PD, dementia, and traumatic brain injury.^14^ These MBI interventions not only support motor rehabilitation but also provide speech comprehension and production, as well as linguistic, cognitive, emotional, and social benefits contributing to holistic recovery.^14^

### Therapeutic potential of MBIs

The dissociation between singing and speaking in individuals with expressive aphasia has long intrigued researchers. Individuals with expressive aphasia often retain the ability to sing lyrics fluently despite impaired speech production.^15^, ^16^, ^17^, ^18^ This phenomenon, along with evidence that music processing predominantly engages the right hemisphere while speech is primarily left‐lateralized, forms the conceptual foundation of MIT,^19^ which is a widely applied therapeutic approach for individuals with nonfluent aphasia.

MIT is widely applied for nonfluent aphasia, particularly applied in right‐handed individuals with left frontal lobe lesions, including Broca's area, and/or damage to the left arcuate fasciculus.^20^ It facilitates speech output using melodic intonation, rhythmic slow verbalization, left‐hand tapping, and formulaic language. MIT thereby initially engages undamaged right hemisphere music‐processing areas, such as right cortico‐striatal networks involved in formulaic language.^21^

However, growing neuroimaging evidence suggests that over the course of therapy, MIT may promote neuroplastic changes not only in the right hemisphere but also in the left hemisphere or bilaterally.^21^, ^22^, ^23^ These findings challenge earlier models and highlight the complexity of its underlying mechanisms. Discrepancies in the literature may stem from small sample size, heterogeneity in lesion location and size, and variations in neuroimaging task designs.^22^, ^23^ To clarify the neural basis of MIT, future research should include structural and resting‐state functional imaging in larger cohorts, with detailed lesion characterization. Including task‐based functional magnetic resonance imaging data from healthy controls may further help differentiate MIT‐induced changes from typical task‐related activations.

Despite current uncertainties regarding its precise neural underpinnings, the demonstrated clinical benefits of MIT, along with the promise of other MBIs, support continued investigation into their use as standalone or adjunct approaches in speech‐language rehabilitation. Future anatomical validation of the association between MBIs and specific brain regions would further strengthen the evidence and advance our understanding of their therapeutic potential.

### Recent systematic reviews and meta‐analyses

Over the past 3 years, five review papers have assessed the efficacy of MBIs for nonfluent aphasia. These include: (1) a systematic review of 26 randomized controlled trials (RCTs) and non‐RCTs;^24^ (2) three systematic reviews with meta‐analyses, which analyzed four RCTs,^25^ six RCTs,^26^ and eleven RCTs,^27^ respectively; and (3) a meta‐analysis combining data from three RCTs and 18 non‐RCTs.^28^

All the meta‐analyses included the same four RCTs, although Liu et al.^26^ and Gu et al.^27^ incorporated additional distinct studies. These reviews consistently observed significant improvements in repetition measured by using standardized assessments, although the sample sizes were small. For instance, Gu et al.^27^ reported moderate quality evidence supporting significant improvements in repetition across nine RCTs, while Liu et al.^26^ noted significant gain in repetition across five RCTs, with individual study quality ratings ranging from acceptable to excellent based on the Physiotherapy Evidence Database scale.^29^ In addition, Liu et al.^26^ demonstrated significant improvements in functional communication across six RCTs (study quality ranging from poor to excellent) and in naming across three RCTs (study quality ranging from acceptable to excellent). However, these significant results might be attributed to the use of mean difference as a summary statistic, whereas other studies utilized standardized mean difference (effect size). Furthermore, Liu et al.^26^ conducted a subgroup analysis, revealing that low‐quality RCTs (*n* = 3) showed significant positive effects in functional communication, whereas high‐quality RCTs (*n* = 3) did not. Despite promising results of MBIs for nonfluent aphasia, several limitations remain in the current evidence. A critical concern is the variability in risk‐bias assessments and methodological differences among meta‐analyses.

To ensure reliable conclusions, high‐quality studies are essential. Of the four meta‐analyses discussed, three employed risk‐of‐bias assessments using The Cochrane Collaboration's tool.^30^

However, a comparison of the assessments for the same four RCTs across these meta‐analyses revealed inconsistencies in their ratings. For example, when evaluating seven bias domains, only two domains were consistently rated as “low risk” across all meta‐analyses for one RCT. Similarly, three domains were consistently rated “low risk” for another RCT, and four domains for two other RCTs. These discrepancies highlight the challenges in synthesizing evidence from studies with varying levels of methodological objectivity. Given the potential of MBIs as evidence‐based interventions for nonfluent aphasia, advancing the field requires rigorous, high‐quality RCTs. Despite promising outcomes in previous studies, significant gaps remain due to methodological inconsistencies and limitations in existing systematic reviews and meta‐analyses, particularly in the assessment of bias. These limitations undermine the reliability of the synthesized evidence and hinder the ability to draw robust conclusions.

### Use of the revised Joanna Briggs Institute critical appraisal tool for risk of bias assessment

The current systematic review addresses methodological inconsistencies in studies examining MBIs for nonfluent aphasia. To ensure a rigorous and outcome‐sensitive evaluation, we employed the revised Joanna Briggs Institute (JBI) critical appraisal tool for RCTs to assess risk of bias. This tool was selected based on several methodological advantages that align with the specific needs of this review. Unlike traditional tools such as the Cochrane Risk of Bias Tool,^30^ which emphasize broader domains like external validity, imprecision, and selective reporting, the revised JBI tool focuses exclusively on internal validity and statistical conclusion validity. This targeted approach minimizes subjectivity by eliminating ambiguous categories such as *other bias* and instead uses 13 specific and standardized questions that improve quality and reproducibility. Crucially, the revised JBI tool extends the assessment of bias beyond the study level to also include outcome and result levels, which is particularly important in the context of MBI studies where outcomes are heterogeneous and effect sizes vary. This updated tool is also aligned with internationally accepted standards for evidence synthesis, including the PRISMA 2020 guidelines^31^ and the GRADE approach.^32^ While recognizing the value of the Cochrane tool, we determined that the revised JBI tool offered the most appropriate and robust framework for evaluating internal validity and inferential strength in this field. Its emphasis on statistical conclusion validity was a decisive factor given the small sample sizes and methodological variability common in studies of MBIs for aphasia.

This methodological approach supports the overarching aims of the current review, which are to systematically examine the current state of evidence on MBIs for nonfluent aphasia by addressing the following key research questions: (1) What types of MBIs have been used for nonfluent aphasia recovery? (2) What are the preliminary conclusions on efficacy of MBIs for nonfluent aphasia recovery? and (3) to evaluate the number of RCTs with low risk of bias and identify the common methodological limitations.

## MATERIALS AND METHODS

This systematic review was conducted in accordance with the JBI methodological framework,^33^ using the Preferred Reporting Items for Systematic Reviews and Meta‐Analyses Extension for Scoping Reviews (PRISMA‐ScR) criteria: Checklist and Explanation guidelines.^34^

### Eligibility criteria

This review included RCTs published in peer‐reviewed journals that assessed the efficacy of MBIs for nonfluent aphasia recovery in individuals aged 19 years or older. Only studies published in English and within 20 years preceding July 2024 were considered for the current review. RCTs were excluded if they did not report pre‐ and post‐speech and language outcomes or if they lacked sufficient methodological details, such as participants’ characteristics, interventional methods, outcome measures, or relevant statistical analyses.

### Search strategy

The comprehensive search was conducted across three major databases like Medline, Pubmed, and APA PsycInfo to identify relevant studies published using PICOS.^35^ The search terms included “music,” “song*,” “melod*,” “beat,” “intonation,” “auditory rhythm*,” “singing or sing” to define musical components used in the interventions combined with the terms “therap*,” “intervention*,” “training,” “treatment*,” or “rehabilitation.” These search results were further combined with the term “aphasi*” to capture all primary clinical populations of interest causing aphasia. The search strategy was developed by two authors (Y.K. and E.T.) in collaboration with a university research librarian to ensure a thorough and systematic approach (Table S1). Additionally, a manual search of reference lists of selected studies was performed to identify any additional relevant articles, ensuring the inclusion of current and emerging evidence.

### Data selection process

The search results were imported into the Covidence systematic review software, and duplicates were removed. Two reviewers (Y.K. and J.B.K.) independently screened the titles and abstracts of the retrieved studies based on the predefined inclusion and exclusion criteria. Full‐text articles of potentially relevant studies were then assessed for eligibility following the same procedure. In cases where conflict or disagreements arose during the screening process, a third independent reviewer (E.T.) was consulted to resolve them.

### Data collection process

The data were charted from included studies, focusing on study design, clinical diagnosis, population characteristics, intervention details, and outcomes measured. Each study was evaluated for its thematic description and quantitative analysis. The first author (Y.K.) created a customized data extraction table to organize the extracted information. The coauthor (P.S.A.) verified the accuracy of the extracted data. All reviewers convened following the initial data extraction to ensure consistency and resolve any discrepancies.

### Data items, outcomes, and prioritization

The following data that were extracted from the studies encompassed several key aspects to ensure a comprehensive review. The author(s) and study design were documented to classify the methodological framework of each study, such as whether it was a parallel or crossover design. Participant characteristics included the clinical diagnosis, total number of participants, gender distribution, and age (mean and standard deviation or an age range). Intervention details were meticulously documented, including the type or technique of MBIs, along with the dose and duration of the intervention. The personnel involved in delivering the interventions were also noted. Primary outcome measures, limited to standardized tools, were listed to evaluate the efficacy of the interventions. Additionally, significant results (*p* <0.05) were extracted and detailed based on pre‐intervention (T1), mid‐intervention (T2), and post‐intervention (T3) time points using “<” or “>” to denote improvement or deterioration.

Data from selected studies were collated, summarized, and reported based on the type of MBIs for nonfluent aphasia: (1) MIT/MMIT and (2) singing‐based interventions. This structured approach ensured a thorough and systematic synthesis of data, providing a clear understanding of the efficacy and scope of various MBIs in the recovery of nonfluent aphasia. The data summarized based on the aforementioned data items primarily focussed on pre‐ and post‐speech and language outcomes, with the rationale of prioritizing interventions with the most statistical results to guide the clinical applications as it directly measures the efficacy of MBIs in improving nonfluent aphasia, which is the focus of the review.

### Risk of bias assessments

To assess the methodological quality and risk of bias of the included RCTs, we employed the revised JBI critical appraisal tool for RCTs.^36^, ^37^ This tool offers a systematic and transparent approach to critical appraisal, aligning with contemporary standards in evidence synthesis. Two reviewers (Y.K. and P.S.A.) independently conducted the assessments, after which the first author charted the results to provide a structured overview of the findings. Any discrepancies identified in their evaluations were resolved through consensus discussions to ensure accuracy and consistency. The assessment focused on key methodological domains with specific questions provided in Table S2.

In line with the approach adopted by Barker et al.,^36^, ^37^ we focused on the first 10 items (Q1–Q10) of the JBI tool, which pertain to internal validity covering essential aspects such as randomization, blinding, group similarity, and outcome measurements. These domains were deemed most relevant to our review objectives, particularly the evaluation of core methodological rigor in MBIs for nonfluent aphasia.

Questions 11–13 of the tool, which relate to statistical conclusion validity and data analysis robustness, were not included in the scoring criteria. This decision was guided by two considerations: (1) consistency with established precedent in recent literature,^36^ which enabled comparisons across reviews; and (2) the need to maintain focus on internal validity, which is a primary determinant of study quality in bias assessments. While we acknowledge the importance of statistical rigor, these aspects were separately considered in our narrative synthesis rather than in the formal scoring system.

Studies were rated according to their total score out of 10: scores of 8–10 indicated “low risk of bias,” 5–7 indicated “moderate risk,” and ≤4 were classified as “high risk.” This grading approach facilitated the identification of high‐quality evidence and enabled a clearer interpretation of the strength and reliability of findings across included studies. The risk of bias assessment thus played a central role in guiding data synthesis and interpretation within this review.

## RESULTS

**FIGURE 1:**
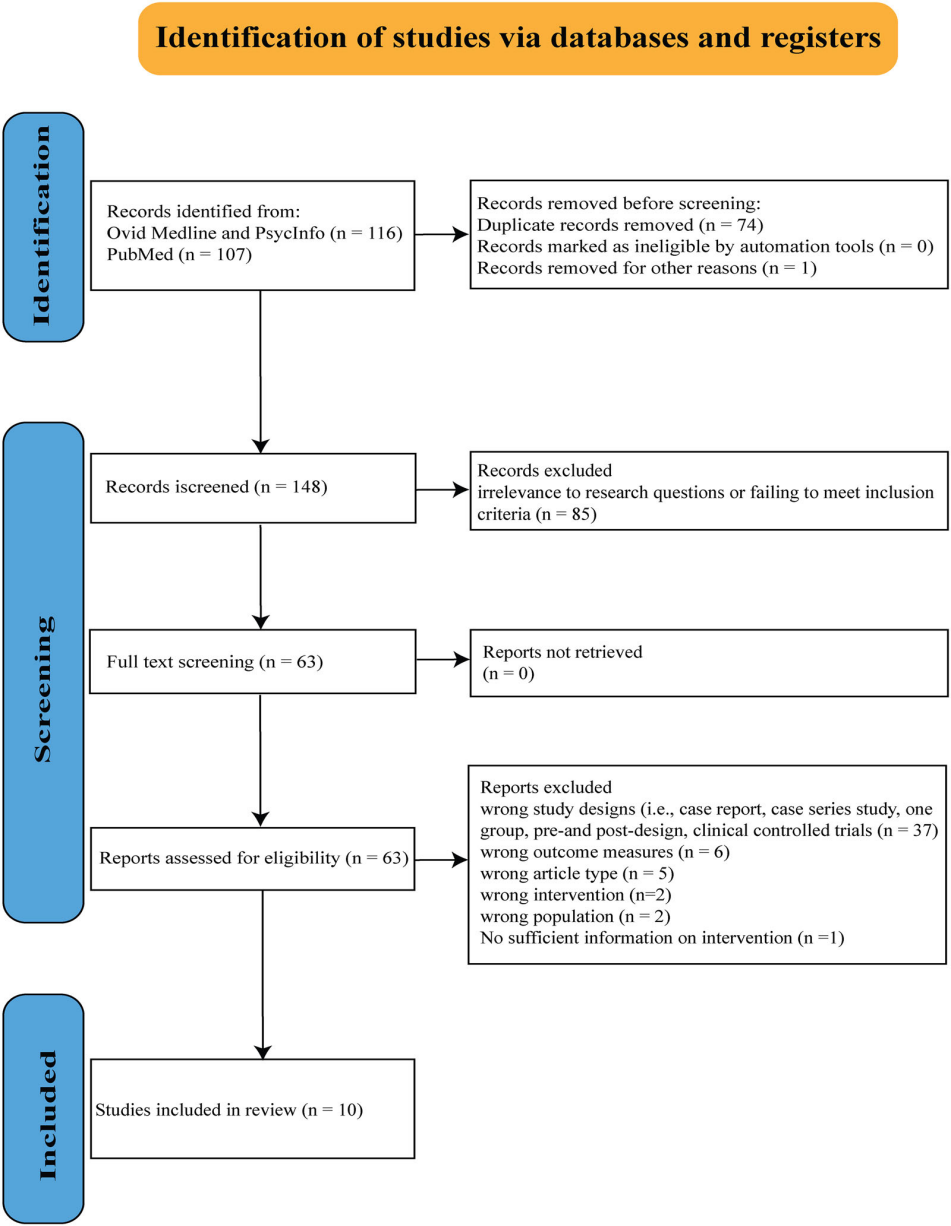
PRISMA‐ScR flow diagram: Selection of sources of evidence.

As illustrated in the PRISMA‐ScR flow chart in Figure 1, a total of 148 studies were identified from searches of three electronic databases. Based on the title and the abstract screening, 85 articles were excluded due to irrelevance to the research questions or failing to meet the inclusion criteria. A total of 63 articles were retained for full‐text review. After a thorough screening, 53 studies were excluded for reasons such as methodological limitations, insufficient data, or lack of relevance to the focus on MBIs for nonfluent aphasia recovery. Consequently, 10 studies were considered eligible in this systematic review and are summarized in Table 1.

**TABLE 1:** Articles assessed in the systematic review.

Authors, year	Study design	Participant characteristics Groups: [1] experimental condition, [2] & [3] control condition(s)				Intervention			Primary outcome measures (standardized only)	Significant results (*p*<0.05; T1: pre‐intervention; T2: mid‐intervention; T3: post‐intervention)
		Diagnosis (*n*)	*n* (male)	Age (yr, mean age ± SD or range)	Major inclusion criteria and/or reported demographic and clinical characteristics	Type/technique	Dose/duration	Personnel involved		
**Nonfluent aphasia (*n*=10)**										
***MIT/MMIT (n=8)***										
**Haro‐Martínez et al., 2019** ^45^	Crossover	Nonfluent aphasia/stroke (*n* = 20)	Grp[1]: 14 (10) Grp[2]: 6 (2)	Grp[1]: 65.2±15.1 Grp[2]: 61.7±13.3	Unilateral LH stroke; >6 mos post‐stroke (median (mos): Grp[1]: 16,Grp[2]: 20.5); previous conventional speech therapy post stroke; resistant to speech therapy; no psychotropic drug use; NS in education btw grps	Individual (Spanish) Grp[1]: A→B Grp[2]: B→A A. MIT B. No intervention	30 min, 2 sessions/ wk for 6 wks	SLP trained in MIT	{1}BDAE (repetition) {2}BDAE (auditory comprehension)	Outcome {1}&{2}: NS
**Van Der Meulen et al., 2016** ^43^	Wait‐list	Nonfluent aphasia/stroke (*n*=23)	T1 Grp[1]: 10 (7) Grp[2]: 7 (3) T2 Grp[1]: 10 (7) Grp[2]: 6 (NA)	T1 Grp[1]: 58.1±15.2 Grp[2]: 63.6± 12.7	1 yr post stroke (mos; Grp[1]: 33.1±19.4, Grp[2]: 42.6±2.7); first unilateral LH stroke; no prior intensive MIT; MIT candidacy assessed using AAT	Individual (Dutch) Grp[1]: A→B Grp[2]: B→A A. MIT B. No intervention	5 h/wk for 1−6 wks (minimum 3 h /wk plus iPod‐based homework); NS in treatment intensity between grps	SLP trained to deliver MIT	{1}SSRT {2}ANEL {3}AAT (naming) {4}AAT (repetition) {5}AAT (auditory comprehension)	Outcome{4}: significant improvement at T2 compared to T1 in Grp[2]
**Van Der Meulen et al., 2014** ^41^	Crossover	Nonfluent aphasia/stroke (*n*=76)	T1 Grp[1]: 16 (4) Grp [2]: 11 (7) T2 Grp [1]: 14 (NA) Grp[2]: 11 (NA) T3 Grp[1]: 14 (NA) Grp[2]: 10(NA)	T1 Grp[1]: 53.1±12.0 Grp[2]: 52.0±6.6	First LH stroke; 2−3 mos post stroke (wks; Grp[1]: 9.3±2.0, Grp[2]: 11.9±5.9); no prior intensive MIT; MIT candidacy assessed using AAT	Individual (Dutch) Grp[1]: A→B Grp[2]: B→A A. MIT B. Conventional treatment for severe aphasia plus homework	5 h/wk for 6 wks	SLP trained to deliver MIT	{1}SSRT {2}ANEL {3}AAT (naming) {4}AAT (repetition)	Outcome {1}: significant improvement at T3 compared to T1 in Grp[1]. {2} to {3}: significant improvement at T2 and T3 compared to T1 in Grp[1]. {4}: significant improvement at T2 compared to T1 in Grp[1]; significant improvement at T3 compared to T1 in Grp[1] & Grp[2].
**Conklyn et al., 2012** ^40^	Parallel	Nonfluent aphasia/acute stroke (*n*=24)	Visit1 (V1) Grp[1]: 14 (6) Grp[2]: 10 (8) Visit2 (V2) Grp[1]: 9 (NA) Grp[2]: 8 (NA)	Grp[1]: 58.3±16.7 Grp[2]: 65.3±11	Mild to severe aphasia on NIHSS; damage to the left middle cerebral artery territory; acute and subacute stroke; any dysarthria noted to be less than their	Individual Grp[1]: MMIT Grp[2]: Conversation	10−15 min, two sessions	Music therapist trained in MMIT	WAB {1}Adjusted total score = (2.5×responsive)+(0.625×repetitive) {2}responsive {3}repetitive	T1 versus T3 at V1 Outcome **{**1}: significant improvement at T3 compared to T1 in Grp[1]; significant improvement in Grp[1] compared to Grp[2]
					aphasia; no receptive aphasia greater than expressive aphasia; expressive aphasia, Broca's type; no dysarthria or AOS					{2}: significant improvement at T3 compared to T1 in Grp[1] (items 2 and 3); significant improvement in Grp[1] compared to Grp[2] (items 2 and 3). V1 versus V2 at T1 Outcome{1}: significant improvement at V2 compared to V1 in Grp[1] & Grp[2]. {2}: significant improvement at V2 compared to V1 in Grp[1]; significant improvement in Grp[1] compared to Grp[2]. {3}: significant improvement at V2 compared to V1in Grp[2].
**Yan et al., 2023** ^42^	Parallel	Nonfluent aphasia/stoke (*n*=39)	Grp[1]: 13 (5) Grp[2]: 13 (10) Grp[3]: 13 (7)	Grp[1]: 63.46±10.187 Grp[2]: 55.46±12.204 Grp[3]: 56.77±9.791	First LH stroke; 1 wk post‐stroke (days; Grp[1]: 241.23±373.78, Grp[2]: 92.23±122.41, Grp[3]: 122.38±299.17); WAB AQ < 93.8; Grp[1]: 24.92±26.58, Grp[2]: 30.138±27.58, Grp[3]: 35.09±37.91; no severe dysarthria	Individual (unspecified Chinese language) Grp[1]: MMIT with tDCS Grp[2]: MMIT with sham stimulation Grp[3]: speech therapy	15 sessions for 3 wks Grp[1]: 20‐min tDCS + 30‐min MIT Grp[2]: 30‐s sham stimulation + 30‐min MIT Grp[3]: 30‐min speech therapy	SLP	WAB {1}AQ {2}spontaneous speech {3}repetition {4}naming {5}comprehension	Outcomes {1} to {4}: significant improvement at T3 compared to T1 in Grp [1] & Grp [2]. {1}: significant improvement at T3 compared to T1 in Grp[1], Grp[2], & Grp[3]. Outcomes {2) & {3}: significant improvement in Grp[1] compared to Grp[3], NS in Grp[1] & Grp[2]. {4}: significant improvement in Grp[1] compared to Grp[2] & Grp[3]. {5}: significant improvement at T3 compared to T1 in Grp[1], Grp[2], & Grp[3]; significant improvement in Grp[1] compared to Grp[2] & Grp[3].
**Zhang et al., 2023** ^38^	Parallel	Different types of nonfluent aphasia/stroke (*n* = 40)	Grp[1]: 20 (16) Grp[2]: 20 (17)	Grp[1]: 50.15±15.44 Grp[2]: 51.6±14.27	LH stroke diagnosed with fMRI or CT; severity assessed on NIHSS; global, Broca's, transcortical mixed, and TMA; >0.5 mos post stroke (mos; Grp[1]: 2.31±1.29, Grp[2]: 1.82±1.39); consistent physical therapy and standard care; no professional music education	Individual (Mandarin) Grp[1]: MMIT Grop[2]: Speech therapy	30‐min, 5 sessions/wk for 4 wks	Grp[1]:Music therapist with NMT training Grp[2]:SLP licensed as rehabilitation therapist	BDAE {1}AQ {2}spontaneous speech {3}listening comprehension {4}repetition {5}naming	Outcomes {1} to {5}: significant improvement at T3 compared to T1 in Grp[1] & Grp[2]. {2}(sum & information): significant improvement in Grp[1] compared to Grp[2]. {5} (sentence completing): significant improvement in Grp[1] compared to Grp[2].
**Zhang et al., 2021** ^39^	Parallel	Different types of nonfluent aphasia/stroke (*n*=40)	Grp[1]: 20 (16) Grp[2]: 20 (15)	Grp[1]:52.90±9.0 Grp[2]:54.05±10.8	LH stroke diagnosed with fMRI or CT; severity assessed on NIHSS; global, Broca's, and TMA; > 15 days post stroke (mos; Grp[1]: 2.57±1.74, Grp[2]: 1.96±1.38); consistent physical therapy, occupational therapy, and routine care; no professional musical experience	Individual (Mandarin) Grp[1]: MMIT Grp[2]: Speech therapy	30 min, 5 sessions/wk for 8 wks	Grp[1]: Music Therapist with NMT training Grp[2]: SLP	BDAE {1}AQ {2}spontaneous speech {3}listening comprehension {4}repetition {5}naming	Outcome {1} to {5}: significant improvement at T3 compared to T1 in Grp[1]. {1},{2}(information), {3},{4}&{5}(spontaneous naming): significant improvement in Grp[1] compared to Grp[2].
**Kiyani et al., 2023** ^44^	Parallel	Broca's aphasia (*n*=50)	Grp[1]: 25(18) Grp[2]: 25(16)	Grp[1]: 60±2.1 Grp[2]: 55±11.7	3 mos post‐aphasia; severe Broca's aphasia; mixed handedness (Grp[1]: left (*n*=3)); no cognitive impairment	Individual (Urdu) Grp[1]: MMIT (in‐person) Grp[2]: Verbal Expressive Skill Management Program in Urdu (Android‐based)	30−45 min, 4 sessions/wk for 16 wks (total 64 sessions)	SLP	BDAE {1}articulatory agility {2}phrase length {3}grammatical form {4}prosody/intonation {5}spontaneous speech {6}word finding {7}repetition {8}auditory comprehension	Outcomes {2}, {3}, & {8}: significant improvement at T3 compared to T1 in Grp[1]. {1} to {8}: significant improvement at T3 compared to T1 in Grp[2]. {1} to {7}: significant improvement in Grp[2] compared to Grp[1].
***Singing (n=2)***										
**Zumbansen et al., 2017** ^47^	Parallel	Aphasia (mild to severe disfluent speech)/stroke (*n*=21) and brain tumor (*n*=1)	Grp[1]: 7 (2) Grp[2]: 8 (3) Grp3[3]: 7 (2)	Grp[1]: 63.4±7.5 Grp[2]: 54.0±19.6 Grp[3]: 54.0±11.6	Chronic aphasia (yrs; Grp[1]: 5.5±5.0, Grp[2]: 4.6±3.8, Grp[3]: 12.8±13.0); no current participation in a speech‐language intervention; mild‐to‐moderate, moderate, and severe aphasia; no dementia or severe nonverbal cognitive deficit	Grp[1]: Choir sessions Grp[2]: Drama classes Grp[3]: No intervention (waitlist)	2 h, 1 session/wk for 26 wks	Grp[1]: Experienced choir leader in aphasia. Grp[2]: Experienced drama teachers in aphasia.	{1}TLDC {2}ABA2 {3}Automated series (MT86) {4}Repetition (MT86 and ABA2) {5}Naming (MT86 and ABA2) {6}Comprehension (MT86)	Outcomes {1} to {6}: NS
**Jungblut et al., 2022** ^46^	Parallel	Different types of nonfluent aphasia/stroke (*n*=22)	Grp[1]: 10 (6) Grp[2]: 10 (7)	Grp[1]: 53.3 (49–70) Grp[2]: 57.2 (51–69)	Post‐stroke (yrs; Grp[1]: 5.3(1–9), Grp[2]: 4.9(1–9)); lesion size (cm^3^; Grp[1]: 117.9, Grp[2]: 134.3); first LH stroke; Broca's (*n* = 5) and global aphasia (*n* = 5)/grp	Individual (German) Grp[1]: SIPARI Grp[2]: Speech Therapy	45 min, biweekly sessions (total of 32 sessions)	Grp[1]: Certified SIPARI therapists Grp[2]: SLP	AAT {1} spontaneous speech {2}repetition {3}naming {4}comprehension {5}profile	Outcome {1} (articulation and prosody), and {2} to {5}: significant improvement in Grp[1] compared to Grp[2]

Abbreviations: AAT, Aachener Aphasie Test; ABA2, Apraxia Battery for Adults‐Second Edition; ANELT, Amsterdam‐Nijmegen Everyday Language Test; AOS, apraxia of speech; AQ, aphasia quotient; BDAE, Boston Diagnostic Aphasia Examination; CT, computed tomography; fMRI, functional magnetic resonance imaging; grp/grps, group(s); h, hour; LH, left hemisphere; min, minutes; MIT, Melodic Intonation Therapy; MMIT, modified Melodic Intonation Therapy; mo/mos, month(s); NA, not available; NIHSS, National Institute of Health Stroke Scale; NMT, Neurologic Music Therapy; NS, not significant; SD, standard deviation; SIPARI, Singing, Intonation, Prosody, Breathing (German Atmung), Rhythm, and Improvisation; SLP, speech language pathologist; SSRT, Sabadel story retelling task; tDCS, transcranial direct current stimulation; TLDC, Test Lillois de Communication; TMA, transcortical motor aphasia; WAB, Western Aphasia Battery; wk/wks, week(s); yr/yrs, year(s).

### Study characteristics

#### MIT/MMIT

Eight RCTs, including two trials with cross‐over designs, investigated the effects of MIT/MMIT on nonfluent aphasia recovery. The sample sizes ranged from 20 to 76 participants, all diagnosed with post‐stroke aphasia. Two studies included multiple aphasia types, such as global, Broca's, transcortical mixed, and transcortical motor aphasia.^38^, ^39^ Four RCTs focused on participants in acute/subacute stages of recovery,^38^, ^39^, ^40^, ^41^ while the remaining studies examined individuals in chronic stages. One RCT included participants at various stages post‐stroke.^42^

 All of the MIT/MMIT interventions were delivered in person. Eight studies were conducted in non‐English languages: two in Mandarin,^38^, ^39^ one in an unspecified Chinese language,^42^ two in Dutch,^41^, ^43^ one in Urdu,^44^ one in Spanish,^45^ and one in German.^46^ Regarding dose and duration for treatment, there are two trials by the same research group who implemented sessions totaling 5 h per week for 6 weeks.^41^, ^43^ Across other studies (six studies), the intervention session durations ranged from 10 to 50 min, with frequencies from twice per week to daily, spanning 2−64 sessions over 2 days to 16 weeks. One study conducted only two sessions, but the interval between them was not specified.^40^

The comparison interventions varied across the trials. Three trials used speech therapy^38^, ^39^, ^42^ and one study lacked a detailed description about the intervention.^39^ Other studies included conventional treatment for severe aphasia that targeted linguistic modalities like writing, language comprehension, nonverbal communication strategies, but did not emphasize spoken output^41^ or conversational interactions with the music therapist regarding participants’ impairment, treatment, outcomes, and issues related with aphasia.^40^ In one trial, MIT (provided during regular therapy sessions, but no description mentioned for MIT) was compared to an Android‐based intervention program called Verbal Expressive Skill Management Program in Urdu, delivered by speech language pathologists (SLPs).^44^ Two trials had comparator groups that received no active intervention.^43^, ^45^ However, one study allowed participants to engage in social interaction and low‐intensity group therapy to support verbal and nonverbal communication, although details were scarce.^43^ However, there was no further information on the patients’ participation.

Interventions were provided by trained SLP, music therapist, or both depending on the trial. Three cross‐over design studies involved SLPs trained in MIT/MITT providing the interventions.^41^, ^43^, ^45^ In addition, in two studies, SLPs delivered MMIT without specific training details.^42^, ^44^ Two studies employed both SLPs and music therapists, providing their respective interventions,^38^, ^39^ while in one study, only a music therapist trained in MMIT was involved.^40^

Outcome measures varied across studies and included a combination of standardized tools. Four studies used the Boston Diagnostic Aphasia Examination (BDAE), including two by the same research group.^38^, ^39^, ^44^, ^45^ Two studies employed the Western Aphasia Battery (WAB), with modifications in one trial to elicit longer responses.^40^, ^42^ Other measures (two studies) included the Aachen Aphasia Test (AAT) and the Amsterdam‐Nijmegen Everyday Language Test (ANELT).^41^, ^43^ Additional tools included Communicative Activity Log (CAL)^45^ and Sabadel story retelling task.^41^, ^43^

#### Singing‐based interventions

Two RCTs used singing‐based interventions in participants with chronic aphasia.^46^, ^47^ Both studies included 22 chronic participants with different types of nonfluent aphasia^43^ and with different types of aphasia including disfluent speech.^47^ Zumbansen et al.^47^ also included one participant with aphasia caused by a brain tumor.

The study by Jungblut et al.^46^ implemented a protocol developed by the primary study author called SIPARI (a rhythmic‐melodic‐based voice training) as the intervention. SIPARI focused on singing, intonation, prosody, breathing, rhythm, and improvisation, with speech therapy as a control intervention. Each intervention session lasted 45 min and was conducted twice weekly for a total of 32 sessions. A certified music therapist and an SLP delivered their respective interventions. The intervention effects were assessed using AAT.

In contrast, Zumbansen et al.^47^ explored the effects of choir singing with two control conditions: drama classes and no intervention. Choir sessions were led by an experienced choir leader, while drama classes were facilitated by two experienced drama teachers. Each session lasted 2 h and was conducted weekly over 26 weeks. Outcome measures included the Test Lillois de Communication, Apraxia Battery for Adults‐Second Edition (ABA2), and MT86.

### Types of MBIs for nonfluent aphasia

The studies included various MBIs to aid recovery from nonfluent aphasia, specifically MIT/MMIT and singing‐based interventions. Out of 10 eligible studies, eight studies employed MIT/MMIT and two used singing‐based interventions.

MIT was the most commonly implemented approach, following a structured, stepwise progression that integrates rhythm and melody to facilitate language recovery. Key elements of MIT included rhythmic hand tapping, intoned speech production, and progression from simple to natural speech. For example, in the Spanish version of MIT, as described by Haro‐Martínez et al.,^45^ patients began with basic phrases and progressed to more complex ones, each reinforced with images for visual context and rhythmic hand tapping to maintain tempo and rhythm. The three levels of MIT in this study involved increasing complexity in both intonation and phrase structure: level 1 used simple phrases with basic intonation, level 2 included moderate complexity with varied intonation, and level 3 included advanced phrases with complex intonation. MIT was adapted in studies by Van Der Meulen et al.,^41^, ^43^ who implemented a structured approach similar to the American MIT manual.^48^, ^49^ The therapy involved short, formulaic phrases, rhythmic hand tapping, joint production with a therapist, and a gradual reduction of therapist support. In their 2016 study, Van Der Meulen et al.^43^ further innovated by developing an iPod application for independent practices at home. MIT was compared with a control group receiving standard speech therapy, where verbal and nonverbal communication was practiced in social settings with written communication and group activities.

MMIT, a tailored approach for nonfluent aphasia that reduces the steps from singing to speech typical in the traditional MIT protocols, follows a similar structured progression, beginning with simple phrases and progressing to more complex utterances. MMIT also incorporates rhythmic hand tapping to assist with motor planning for vocalization, as seen in the work of Conklyn et al.^40^ This approach includes a gradual transition from singing phrases to natural speech, incorporating both melody and rhythm to activate speech abilities and cognitive functions. In addition, Yan et al.^42^ combined MMIT with transcranial direct current stimulation (tDCS) to enhance outcomes. Their study included 15 sessions of MMIT combined with tDCS using rhythmic hand tapping at word and sentence levels and transitioning from melodic intonation to natural speech. Similarly, Zhang et al.^38^, ^39^ utilized MMIT with different musical tools, including keyboard or guitar, and applied a structured approach focusing on rhythmic chanting and intonation, transitioning from simple phrases to more complex sentences. Lastly, Kiyani et al.^44^ conducted a study with a more extended MIT protocol, demonstrating the effectiveness of MIT in conventional speech therapy contexts.

The current systematic review also identified singing‐based interventions as effective for nonfluent aphasia rehabilitation, with two notable studies highlighting their benefits. Zumbansen et al.^47^ implemented a choir singing intervention focusing on improving articulation, phonetic production, and melodic patterns by facilitating rhythm and synchrony, which enhanced speech coordination and timing. In contrast, Jungblut et al.^46^ introduced the SIPARI (Singing, Intonation, Prosody, Breathing, Rhythm, and Improvisation) approach within an MMIT framework and involved tasks such as sublexical vowel and consonant‐vowel combinations, rhythmic grouping of syllables, and the gradual transition from words to phrases. The approach not only targeted speech production but also focused on cognitive functions like working memory and attention to enhance the retention of auditory and rhythmic information. Both studies aimed to demonstrate the effectiveness of singing‐based interventions by integrating melody, rhythm, and cognitive functions to improve speech and communication in individuals with nonfluent aphasia.

All studies tried to emphasize the adaptability and effectiveness of MBIs, particularly MIT, MMIT, and singing‐based approaches, in nonfluent aphasia rehabilitation. By combining rhythm, melody, and structured progression, these interventions aimed to address both linguistic and cognitive deficits and to demonstrate a promising avenue for enhancing language recovery in individuals with nonfluent aphasia.

### Methodological limitations of MBIs and risk of bias assessment

**FIGURE 2:**
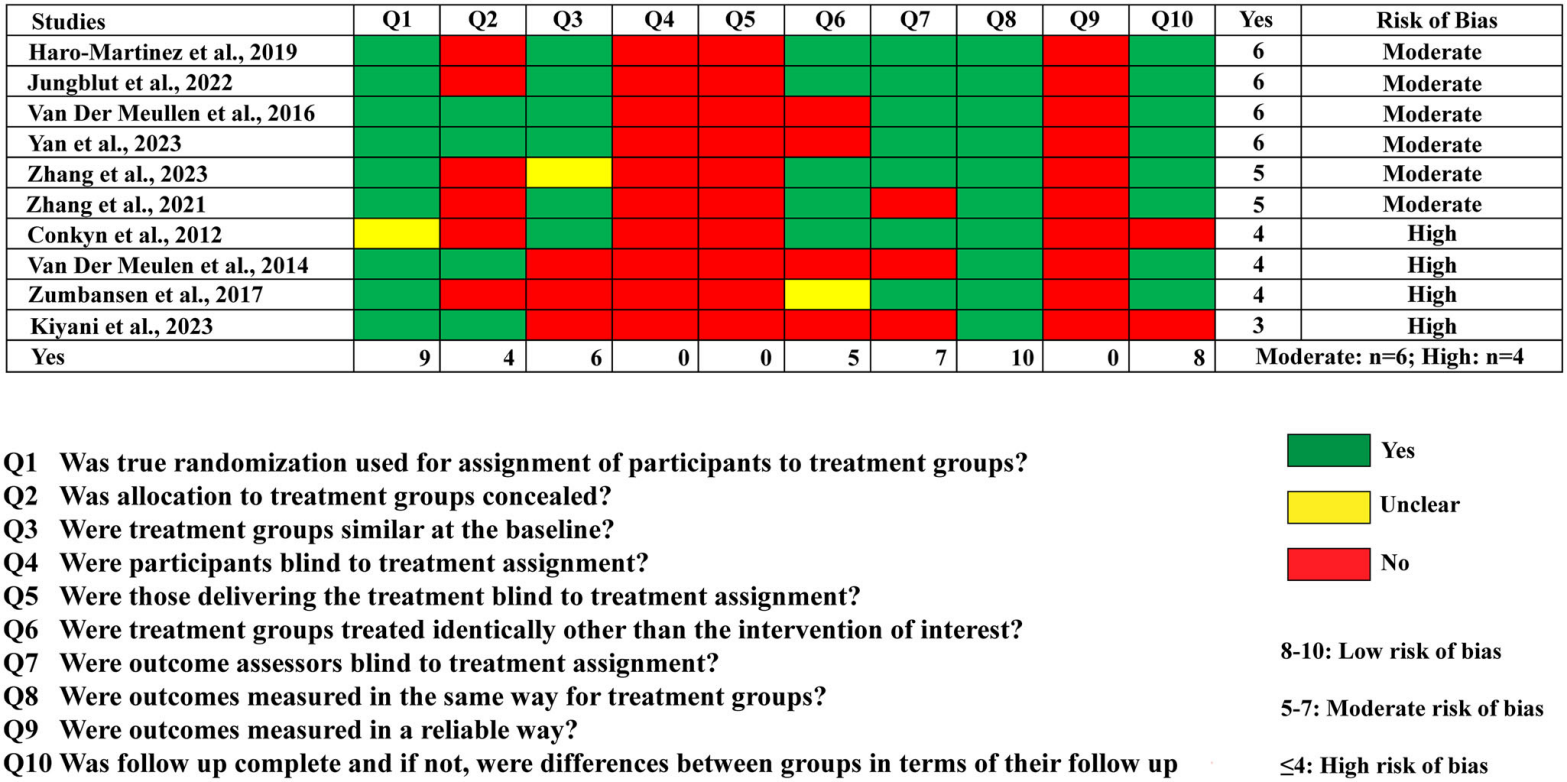
Results of Risk of Bias Assessment using the JBI critical appraisal tool for randomized control trials.

The results of the risk of bias assessments are presented in Figure 2. The overall scores of each study ranged from 3 to 6, reflecting moderate to high risk of bias across the reviewed studies. Among the studies, six demonstrated moderate risk, while four exhibited high risk of bias. No RCT with a low risk of bias has been conducted to date to our knowledge.

Behavioral intervention studies inherently pose challenges for blinding, which contributed to “No” ratings for Q4 (Were participants blind to treatment assignment?) and Q5 (Were those delivering the treatment blind to treatment assignment?) in all trials. Additionally, none of the studies fulfilled Q9 (Were outcomes measured in a reliable way?), as they failed to report essential information regarding the number of raters, their training, or the intra‐rater and the inter‐rater's reliability within the study.

Over half of the studies did not conceal the group allocation (Q2), or half of the studies did not ensure identical treatment across groups outside of the intervention (Q6). For Q6, common reasons for “No” ratings included variability in intervention regimes, lack of clarity on participants’ access to standard care or other treatments, and differing methods of intervention delivery.

**FIGURE 3:**
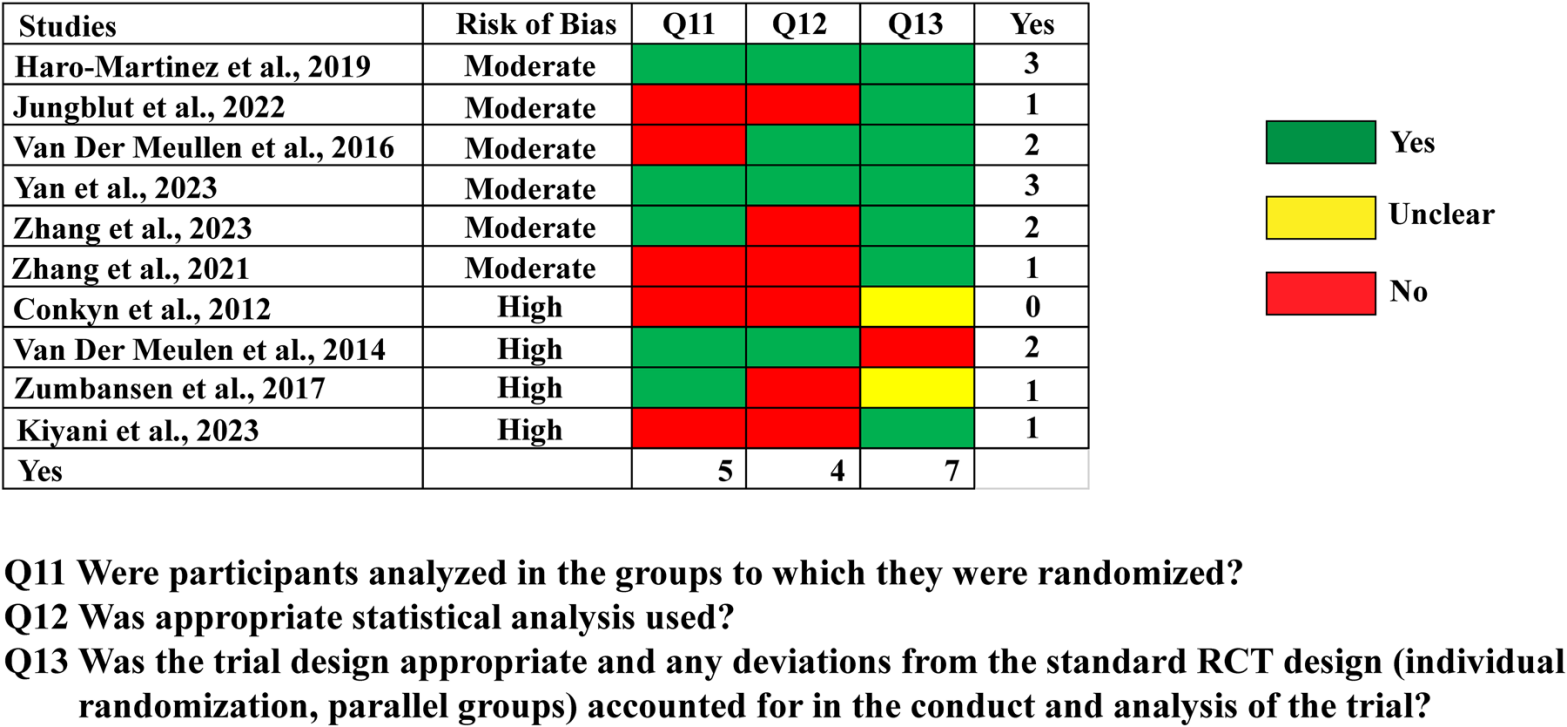
Results of Statistical Conclusion Validity Assessment using the JBI critical appraisal tool for randomized control trials.

The statistical conclusion of the studies was evaluated using three criteria, as summarized in Figure 3. Among the 10 reviewed studies, only two met all the criteria for appropriate statistical practices. Only half of the studies report that the data were analyzed using an intention‐to‐treat (ITT) analysis (Q11). Six out of 10 studies did not use appropriate statistical analyses (Q12), either omitting power analyses or neglecting to report effect sizes. These omissions undermine the robustness of the statistical conclusions drawn from the studies.

### Efficacy of MBIs

The efficacy of MBIs was assessed for various speech and language domains, including automatic speech, spontaneous speech, auditory comprehension, repetition, naming, and verbal communication. The results are presented separately for studies with moderate risk of bias and high risk of bias, as detailed in Table 2, highlighting both within‐ and between‐group analyses to illustrate significant intervention–time interactions. Within‐group analysis showed consistent efficacy of MBIs across various speech and language domains. Significant improvements were observed in repetition in four out of seven studies, including three out of four moderate risk‐of‐bias studies. Three out of four moderate risk‐of‐bias studies also reported significant improvements in information content. In fluency, four out of six studies, including three out of four moderate risk‐of‐bias studies, reported enhancements. Naming abilities also showed progress, with five out of six studies, including three out of four moderate risk‐of‐bias studies, demonstrating significant improvements. Additionally, four out of seven studies, consisting of three moderate risk‐of‐bias and one high risk‐of‐bias studies, found significant improvement in auditory comprehension.

**TABLE 2:** The number of studies demonstrating significantly greater improvement (*p* <0.05) using standardized outcome measures following MBIs: Between‐group and within‐group analysis results.

Risk of bias	Study	Between/within analyses	Automatic speech	Spontaneous speech		Auditory comprehension	Repetition	Naming	Functional communication
				Fluency	Information content				
**Moderate**	**Haro‐Martinez et al., 2019** ^45^ **(MMIT vs. No intervention)**	Between	−	−	−	No (BDAE)	No (BDAE)	−	−
		Within	−	−	−	−	−	−	−
	**Van Der Meulen et al., 2016** ^43^ **(MIT vs. No intervention)**	Between	−	No (SSRT)	No (SSRT)	No (AAT)	No (AAT)	No (AAT)	No (ANEL)
		Within	−	No (SSRT)	No (SSRT)	No (AAT)	No (AAT)	No (AAT)	No (ANEL)
	**Yan et al., 2023** ^42^ **(MMIT + sham stimulation vs. Speech therapy)**	Between	−	No (WAB)	No (WAB)	No (WAB)	No (WAB)	No (WAB)	−
		Within	−	Yes (WAB)	Yes (WAB)	Yes (WAB)	Yes (WAB)	Yes (WAB)	−
	**Zhang et al., 2021** ^39^ **(MMIT vs. Speech therapy)**	Between	−	No (BDAE)	Yes (BDAE)	Yes (BDAE)	Yes (BDAE)	Yes (BDAE)	−
		Within	−	Yes (BDAE)	Yes (BDAE)	Yes (BDAE)	Yes (BDAE)	Yes (BDAE)	−
	**Zhang et al., 2023** ^38^ **(MMIT vs. speech therapy)**	Between	−	No (BDAE)	Yes (BDAE)	No (BDAE)	No (BDAE)	Yes (BDAE)	−
		Within	−	Yes (BDAE)	Yes (BDAE)	Yes (BDAE)	Yes (BDAE)	Yes (BDAE)	−
	**Jungblut et al., 2022** ^46^ **(SIPARI vs. speech therapy)**	Between	−	Yes (AAT: prosody and articulation)	−	Yes (AAT); No (AAT: token test)	Yes (AAT)	Yes (AAT)	−
		Within	−	−	−	−	−	−	−
	**# of moderate risk‐of‐bias studies reporting significant improvement/# of moderate risk‐of‐bias studies testing the domain**	***Between***	***0/0***	***1/5***	***2/4***	***2/6***	***2/6***	***3/5***	***0/1***
		***Within***	***0/0***	***3/4***	***3/4***	***3/4***	***3/4***	***3/4***	***0/1***
**High**	**Conklyn et al., 2012** ^40^ **(MMIT vs. Conversation)**	Between	−	−	−	−	No (modified WAB)	Yes (modified WAB)	−
		Within	−	−	−	−	No (modified WAB)	Yes (modified WAB)	−
	**Van Der Meulen et al., 2014** ^41^ **(MIT vs. Conventional treatment)**	Between	−	No (SSRT)	−	−	No (AAT)	No (AAT)	No (ANEL)
		Within	−	Yes (SSRT)	−	−	Yes (AAT)	Yes (AAT)	Yes (ANEL)
	**Kiyani et al., 2023** ^44^ **(MMIT vs. Verbal Expressive Skill Management Program)**	Between Within	No (BDAE) No (BDAE)	No (BDAE) Yes (BDAE: phase length and grammatical form); No (BDAE: articulatory agility, prosody/intonation, word finding)	− −	No (BDAE) Yes (BDAE)	No (BDAE) No (BDAE)	− −	− −
	**Zumbansen et al., 2017** ^47^ **(Singing vs. drama and no intervention)**	Between Within	− −	No (ABA2; TLDC) −	− −	No (MT86; TLDC) −	No (MT86; ABA2) −	No (MT86; ABA2; TLDC) −	− −
	**# of high risk‐of‐bias studies reporting significant improvement/# of high risk‐of‐bias studies testing the domain**	***Between*** ***Within***	***0/1*** ***0/1***	***0/3*** ***1/2***	***0/0*** ***0/0***	***0/2*** ***1/1***	***0/4*** ***1/3***	***1/3*** ***2/2***	***0/1*** ***1/1***
**Total # of studies reporting significant improvement/total # of studies testing the domain (i.e., moderate and high risk‐of‐bias studied combined)**		**Between** **Within**	**0/1** **0/1**	**1/8** 4/6	**2/4** 3/4	**2/8** 4/5	**2/10** 4/7	**4/8** 5/6	**0/2** 1/2

*Note*: Bold italics indicate the subtotal for studies with moderate or high risk of bias. Bold indicates the overall total across all included studies.

Abbreviations: −, domain not tested by the study; AAT, Aachener Aphasie Test; ABA2, Apraxia Battery for Adults‐Second Edition; ANEL, Amsterdam‐Nijmegen Everyday Language Test; BDAE, Boston Diagnostic Aphasia Examination; MIT, Melodic Intonation Therapy; MMIT, modified Melodic Intonation Therapy; SIPARI, Singing, Intonation, Prosody, breathing (German Atmung), Rhythm, and Improvisation; SSRT, Sabadel story retelling task, TLDC, Test Lillois de Communication; WAB, Western Aphasia Battery.

In contrast, between‐group analysis revealed more varied results. Repetition was assessed by all studies. However, only two of the studies with moderate risk of bias showed significantly better repetition scores for the MBI group compared to controls, while four of eight studies, including three out of four moderate risk of bias, reported significant improvements in naming. Two out of eight studies, including two out of six moderate risk‐of bias studies, demonstrated better auditory comprehension in MBI groups, and only one out of eight studies, including one out of five moderate risk‐of‐bias studies, found significant between‐group differences in fluency. These findings highlight the potential of MBIs, particularly in enhancing repetition and naming abilities, though outcomes for other domains were less consistent. However, it is encouraging that within‐subjects analyses showed significant improvements primarily in moderate risk‐of‐bias studies across various domains. Additionally, MBIs using MMIT and SIPARI led to significantly greater improvements in repetition and naming domains, even when compared to speech‐language therapy.

### Effectiveness of MBIs

Only two studies by the same research group evaluated real‐world communication using the ANELT.^41^, ^43^ In the earlier study, which was rated as having a high risk of bias, the MIT group showed significant improvement in functional communication; however, no significant difference was found compared to the control condition.^41^ In the later study, which was rated as having a moderate risk of bias, the MIT group did not show any significant post‐intervention improvement or significant difference from the control condition in functional communication. MIT may be effective for the recovery of real‐world communication in subacute nonfluent aphasia.

## DISCUSSION

This systematic review conducted a review of current literature on MBIs for recovery of nonfluent aphasia, identifying available interventions for nonfluent aphasia, and it focused on the quality of RCTs and identifying methodological limitations for future improvements. Our search identified 10 RCTs, which were evaluated using the revised JBI critical appraisal tool to assess the risk of bias. None of the RCTs demonstrated a low‐risk bias. Among the studies, eight utilized MIT or MMIT, and two focused on singing‐based interventions. Six RCTs were rated as having a moderate risk of bias, while four were rated as high risk. Common methodological shortcomings included insufficient information on a reliable outcome measurement, lack of group allocation concealment, unequal treatment of groups, and the absence of power analyses and effect size reporting. In the following subsections, we further discuss the types and efficacy of MBIs and methodological limitations in detail, both within the context of the JBI criteria and additional methodological concerns.

### Types of MBIs for nonfluent aphasia

MBIs encompass a range of approaches tailored to improve speech and language functions. Individual MIT/MMIT approaches have been the most commonly used MBIs, featured in eight out of 10 studies. A structured, stepwise progression from intoned to natural speech is a hallmark of MIT. Studies like Haro‐Martínez et al.^45^ and Van Der Meulen et al.^41^, ^43^ highlight improvements in spontaneous speech, repetition, and naming using elements like rhythmic hand tapping and gradual reduction in the therapist's support. Adaptations to traditional MIT include broader pitch ranges, integration with neuromodulation techniques (e.g., tDCS), and culturally specific modifications. Yan et al.^42^ and Zhang et al.^38^, ^39^ reported applications to spontaneous speech and functional communication using Mandarin‐adapted MMIT and MMIT with tDCS. Additionally, studies like Kiyani et al.^44^ and Zhang et al.^38^ underscore the importance of language and cultural adaptations for Urdu and Mandarin speakers, respectively, ensuring relevance and accessibility across populations.

Although MIT/MMIT follows a relatively standardized therapeutic protocol, guidelines for dosage remain unspecified. Consequently, the intervention dosage varied significantly. Except for one study that conducted only two 10‐ to 15‐min sessions, most studies implemented multiple weekly sessions, with each session lasting 20−45 min over a period of 3−16 weeks. Additionally, while MBIs were conducted by MBI‐trained professionals in eight studies, MMIT was delivered by an SLP in two studies. These variations in intervention delivery significantly impact treatment outcomes. Another structured intervention, SIPARI—categorized under singing‐based interventions—incorporated MIT principles and was delivered by a trained therapist. To date, only one RCT has evaluated its efficacy,^46^ highlighting the need for further research of this therapeutic technique.

Lastly, one study investigated a choir intervention,^47^ which differed from others in that it was delivered to a group and led by a choir leader rather than a therapist. The intervention dosage also varied significantly, consisting of 2‐h weekly sessions over 26 weeks. On the one hand, group‐based MBIs, such as choral singing^47^ and drama‐integrated intervention,^46^ have been shown to facilitate language recovery while promoting social interaction and emotional engagement. However, this study did not specify how the intervention directly addressed speech‐language impairments.

### Efficacy of MBIs for nonfluent aphasia

MBIs have emerged as therapeutic tools for addressing the multifaceted challenges of nonfluent aphasia recovery. These interventions employ musical components such as melody, rhythm, and pitch‐based intonation to facilitate speech production and enhance communication abilities. Among various types of MBIs, MIT is the most extensively studied.

The efficacy of MBIs spans several domains crucial for aphasia recovery. In terms of speech production, MBIs have consistently shown improvements in fluency and the informational content of speech. Jungblut et al.^46^ reported that group singing helps transition patients from intoned phrases to spontaneous speech, while Zhang et al.^38^, ^39^ observed significant gains in fluency through structured MMIT protocols. Similarly, Kiyani et al.^44^ highlighted enhanced articulation and speech fluency in culturally adapted interventions. Functional communication, a critical aspect of nonfluent aphasia therapy, has also benefited significantly from MBIs.

Van Der Meulen et al.^41^ emphasized that MIT improved real‐world communication, enabling patients to express needs and engage in meaningful social interactions. Beyond speech production, MBIs have positively impacted auditory comprehension, repetition, and naming. Studies by Jungblut et al.,^46^ Yan et al.,^42^ and Zhang et al.^38^, ^39^ demonstrated improvements in these domains, underscoring the role of rhythmic and melodic cues in aiding language processing. Van Der Meulen et al.^41^ further showed that repetition tasks benefited from structural intonation protocols, reinforcing the therapeutic utility of MBIs. Additionally, MBIs have shown benefits in reducing social isolation and enhancing emotional engagement, as highlighted by Zumbansen et al.,^47^ particularly in group‐based interventions.

MBIs engage intact right‐hemispheric neural circuits to compensate for left‐hemisphere language deficits, thereby facilitating neuroplasticity. While early models of MIT emphasized right‐hemisphere involvement in supporting speech recovery, more recent evidence suggests that neuroplastic changes may also occur bilaterally or within perilesional areas in the left hemisphere. For instance, Yan et al.^42^ emphasized the role of tDCS in enhancing cortical excitability during MMIR, supporting the idea that MBIs can promote activity in language‐relevant brain regions. Although our review only included RCTs, previous neuroimaging studies (e.g., Van de Sandt‐Koenderman et al.^50^) have highlighted significant individual variability in MIT‐induced brain activation, indicating that neural mechanisms may differ based on lesion, severity, and individual responsiveness. These interventions not only improve trained behaviors but also generalize to untrained speech and language tasks, as demonstrated by gains in spontaneous speech in Zhang et al.^39^ Overall, MBIs represent a promising and neurobiologically grounded approach for the rehabilitation of nonfluent aphasia.

These findings suggest that structured MBIs such as MIT/MMIT and SIPARI have great potential for recovery of nonfluent aphasia and may be combined with a group activity such as choral singing to address nonspeech language difficulties associated with the condition. However, this review revealed that the beneficial effects were diminished when MBIs were compared with control conditions, highlighting the need to address serious methodological limitations in current studies to build a high‐level evidence base.

### Effectiveness of MBIs for nonfluent aphasia

One of the two studies demonstrating significant improvement in functional communication following MIT involved participants with subacute stroke. The control group received conventional therapy for severe nonfluent aphasia, which did not emphasize speech production but instead focused on writing, language comprehension, and nonverbal communication.^41^ This study was rated as having a high risk of bias, particularly due to its cross‐sectional design and other concerns discussed in the next section. Therefore, its findings should be interpreted with caution. The other study, which reported no significant improvements, included participants with chronic stroke, and the control group did not receive any intervention.^43^ Given the limited number of studies and their methodological differences, it remains difficult to draw firm conclusions about the transfer effects of MIT to real‐world communication. Further high‐quality research in this area is urgently needed.

### Methodological limitations of MBIs and risk of bias assessment

#### Trial design (cross‐over design)

According to the JBI checklists, a cross‐over design must meet two critical criteria: participants should have a chronic and stable condition where the intervention produces short‐term effects (e.g., symptom relief), and there should be an appropriate washout period between treatments. Among the two cross‐over RCTs reviewed, one study with a high risk of bias included subacute participants,^41^ whereas another with a moderate risk of bias involved chronic participants.^45^ The study with subacute participants did not provide clarity on the appropriateness of the washout periods for MBI and control intervention, potentially obscuring the true intervention effects; therefore, the carryover effects may compromise the validity of the control condition as a true baseline.

A robust cross‐over design should focus exclusively on chronic aphasic patients resistant to standard speech‐language therapy, with a control group receiving no intervention, as exemplified by Haro‐Martínez et al.^45^ Trials employing such design could offer valuable insights into whether MBIs are a viable alternative to conversational speech‐language therapy for individuals with chronic aphasia.

#### Baseline characteristics of participants

For robust trial designs, the participants’ clinical characteristics must be explicitly defined within the inclusion and exclusion criteria and reported in detail. MIT, originally developed for English‐speaking individuals with nonfluent aphasia, targets individuals with profound impairments in language production, poor verbal agility, difficulty in repeating sentences, and relatively preserved auditory speech comprehension.^49^, ^51^ Thus, stringent screening processes are essential to identify suitable candidates for MIT and MMIT.

To enhance trial validity, quantifiable measures of language function should form part of the inclusion and exclusion criteria, such as clearly defined cut‐off scores for auditory comprehension. Additionally, trials should specify or report the presence and severity of co‐occurring conditions such as dysarthria, apraxia, and dysphagia. Other participant characteristics, including cognition, mood, depression, and fatigue, must also be considered, as they can significantly impact intervention outcomes.^52^

While some studies focused on participants in specific stroke recovery stages or aphasia severities and types, others included individuals with diverse clinical profiles. In such cases, the composition of clinical characteristics in both experimental and control groups, as well as group mean scores, must be reported to ensure comparability. Alternatively, statistical adjustments should be implemented to account for variability. Adhering to these steps is crucial to facilitate the derivation of precise and reliable conclusions regarding the efficacy of MBIs.

#### Experimental and control conditions

The control conditions across the reviewed trials varied considerably, ranging from no intervention or standard care to activities unrelated to speech‐language production and therapy. In some studies, disparities in the length and content of intervention across groups raised concerns about comparability. For example, Yan et al.^42^ conducted a trial with a moderate risk of bias in which the MMIT intervention combined with tDCS lasted 50 min per session, MMIT with sham stimulation lasted 30.5 min, and speech therapy without sham stimulation lasted 30 min per session. This variation in intervention duration potentially affected the validity of comparisons between groups.

Similarly, a study by Van Der Meulen et al.^43^ with a moderate risk of bias employed two different delivery methods: in‐person sessions and a home‐based practice via an iPod application. However, the study did not report the time participants spent using each delivery format, leaving it unclear whether the intervention doses were comparable across groups.

Furthermore, three studies indicated that standard care, including speech therapy, was available to all participants during the trial (Table 1). However, most of these studies did not report the extent to which participants engaged in standard care during the intervention period. This lack of documentation introduces uncertainty about whether the groups were treated equally aside from the intervention being evaluated.

In stroke rehabilitation, where standard care is routinely available, it is crucial to specify and quantify the types and levels of participation in such care (see Siponkoski et al.^53^) Control conditions must be carefully selected to accurately assess the efficacy of MBIs. Ideally, MBIs should demonstrate outcomes that are at least equivalent to or surpass those of conventional speech‐language therapy, which remains the gold standard for aphasia rehabilitation.^54^

#### Outcome measures and assessment

The reviewed studies used varied linguistic outcome measures, including standardized tools like the WAB‐R, BDAE, and AAT. While these assessments share similar subtests, repetition was the only measure consistently assessed and reported across studies. Though the focus of the intervention may determine the outcomes reported, it is vital to include measures beyond speech production, such as auditory comprehension, which is critical for both intervention and recovery. Notably, eight out of the 10 studies reported auditory comprehension outcomes.

Standardized assessments such as the BDAE and AAT, while providing normative data, allow for the evaluation of clinically significant improvements at the individual level. To enhance consistency and comparability across studies, standardized outcome measures like the WAB‐R should be routinely employed, as recommended for phase I–IV studies.^55^ Such standardization can enable better evaluation of MBI efficacy and facilitate analysis of the relationships between intervention response, dosage, and patient characteristics.

In addition to traditional speech and language outcomes, it is essential to assess functional communication that reflect the real‐world application of communication skills. Among the reviewed studies, only two studies evaluated functional communication using ANELT.^41^, ^43^ As emphasized by Doedens and Metayard,^56^ the ultimate goal of aphasia rehabilitation is to restore communication in daily life. However, there is no consensus on how much to measure these improvements, which highlights a critical gap in current research. Future studies should incorporate assessments of both speech‐language abilities and functional communication and adapt interventions, such as MIT, to better target functional communication outcomes.

Timing of assessments also varied among the trials. Only one study performed pre‐ and post‐assessments immediately before and after each intervention session,^40^ while others did not specify the timing of assessments. For robust evaluation, future trials should clearly detail the planned timing of assessments in the methods section and ensure these are conducted as close to the intervention period as possible. Reporting whether assessments were carried out as planned for all participants is also essential to maintain methodological rigor.

#### Statistical analysis

The CONSORT guidelines for RCT reporting recommend the use of ITT analysis, which was reported in five of the reviewed studies. However, ITT does not specifically estimate the effects of receiving the intervention of interest.^33^ Since the primary objective of the reviewed trials was to evaluate the efficacy of MBIs, incorporating additional analyses such as treated or per‐protocol could complement ITT by providing insights into the intervention's direct effects, particularly in cases of participant withdrawal. Among the reviewed studies, there was only one conducted per‐protocol analysis.^45^

Of the 10 studies, seven provided data for both within‐ and between‐group analyses. While the selection of statistical tests varied based on factors like data distribution and covariates, robust methodologies such as mixed‐effects models that account for participants as random effects could enhance the reliability and precision of estimates. Employing such approaches in future studies could strengthen the validity of findings by accommodating for individual variability and repeated measures over time.

#### Improvement of study blinding

Although full blinding is inherently challenging in behavioral intervention studies, several methodological strategies can be employed to minimize the risk of bias. As recommended by Mataix‐Cols and Andersson,^57^ researchers should consider concealing the study hypothesis and the number of treatment arms from both participants and therapists when ethically permissible. Recruitment materials should remain neutral in language (“individualized speech‐language therapy for chronic aphasia”) to minimize expectancy effects. Critically, outcome assessors must remain blinded to group allocation and assessment time points, and any discussions between assessors and participants regarding therapy must be strictly prohibited.^57^ These blinding procedures should be prespecified in study protocols and transparently reported to enhance the methodological rigor of future RCTs.

### Recommendations for future RCTs of MBIs

To strengthen the evidence supporting MBIs as either standalone or adjunctive treatment for nonfluent aphasia, future RCTs should incorporate several key methodological improvements and standardized approaches. First, participant selection must be carefully aligned with the intervention criteria to ensure that individuals included in the trials, such as those with nonfluent aphasia, are appropriate candidates for specific MBIs like MIT or MMIT based on their clinical and neuroanatomical profiles (as discussed in “Baseline characteristics of participants”). Second, trials should adopt robust RCT designs, including either parallel‐group or cross‐over designs, and must ensure the consistent delivery of intervention protocols. This includes clearly defined dosage, intensity, and fidelity checks, with interventions administered by trained speech‐language therapists to help isolate the specific active components of MBIs, namely, rhythmic and melodic elements. Third, outcome assessments could include both standardized language assessments and ecologically valid measures of communication to capture not only linguistic improvements but also real‐world communicative efficacy. The inclusion of patient‐reported outcomes and caregiver observations could further enhance the clinical relevance of findings. Finally, to advance understanding of the underlying mechanisms, future studies should incorporate neuroimaging data, particularly structural and resting state functional MRI, to identify neural predictors of response and to map the neuroplasticity associated with MBI‐induced behavioral gains. This multimodal approach will help link behavioral outcomes with neurobiological substrates, ultimately contributing to the optimization and individualization of treatment protocols.

## CONCLUSIONS

This systematic review revealed beneficial research evidence for MBIs applied to aphasia rehabilitation as well as notable methodological limitations, with no RCTs rated as having a low risk of bias. Combined with additional quality concerns and considerable variability in study designs, these methodological limitations complicate drawing definitive conclusions about the efficacy of MBIs for nonfluent aphasia recovery. This is particularly relevant for interventions like MIT, which are widely used clinically and supported by evidence from neuroimaging studies regarding their underlying neural mechanisms. High‐quality RCTs with more standardized designs and well‐defined participant characteristics are essential to establish robust evidence for clinical outcomes evidence. Future RCTs should utilize available assessment tools, such as the JBI appraisal criteria, to guide trial design and improve study quality.^58^ Addressing these challenges will enhance the evidence and further validate the role of MBIs in nonfluent aphasia rehabilitation.

This systematic review has several methodological limitations that may affect the generalization and strength of its conclusions. First, the literature search was restricted to studies published in English, which may have led to language bias and the exclusion of relevant non‐English publications. Second, the search strategy involved a limited number of databases, which might have resulted in the omission of relevant studies not indexed in those sources. Third, although this review focused on RCTs, the number of available RCTs investigating MBIs for nonfluent aphasia was relatively small and included studies that varied widely in terms of sample size, intervention protocols, outcome measures, and duration of follow‐up. This heterogeneity precluded the possibility of conducting a meta‐analysis and limited the ability to draw firm quantitative conclusions. Fourth, to ensure the inclusion of trials with more robust methodological designs, we restricted the search to the past 20 years, which coincided with the widespread use of formal risk‐of‐bias assessments that are inherently subjective. We minimized this by employing a validated tool (the revised JBI critical appraisal checklist) and ensuring inter‐rater consensus. Lastly, the review did not include gray literature, unpublished trials, or trial registry searches, potentially introducing publication bias. Despite these limitations, the review provides a critical and structured overview of the current state of evidence, highlights key gaps in methodology, and informs future directions for designing high‐impact RCTs in this area.

Though there is consistent evidence that MBIs appear to be beneficial for nonfluent aphasia intervention, serious challenges such as variability in responses, small sample sizes, and lack of protocol standardization need to be addressed. Future research should aim to establish standardized methodologies, explore long‐term outcomes, and investigate the generalization of the therapeutic gains to untrained language tasks. Moreover, cross‐cultural studies are essential to optimize interventions for diverse populations, as demonstrated by Zhang et al.^38^ and Kiyani et al.^44^ It is also vital to study the adaptability of these interventions for rehabilitation settings in low‐ and middle‐income countries (e.g., community‐based rehabilitation), ensuring feasibility and effectiveness in such contexts. Despite these challenges, the transformative potential of MBIs highlights their critical value as a core component of nonfluent recovery programs.

## AUTHOR CONTRIBUTIONS

Conceptualization: Y.K., P.S.A., and M.H.T.; Data curation and synthetization: Y.K., E.T., and J.B.K.; Funding acquisition: M.H.T. and P.S.A.; Design of methodology: Y.K. and P.S.A.; Project administration: Y.K. and M.H.T.; Supervision: M.H.T.; Validation: P.S.A. and M.H.T.; Data presentation: Y.K. and P.S.A.; Writing—original draft: Y.K.; Writing—review and editing: P.S.A., E.T., J.B.K., and M.H.T.

## COMPETING INTERESTS

The authors declare no competing interests.

## PEER REVIEW

The peer review history for this article is available at https://publons.com/publon/10.1111/nyas.15387.

## Supporting information




**TABLE S1** Search strategy used for selection of articles in MEDLINE, PsycInfo, and Pubmed.

**TABLE S2** JBI risk of bias assessment and statistical conclusion validity questions.

## Data Availability

Data sharing is not applicable to this article as no datasets were generated or analyzed during the current study.
