# Supplementary material for: Music‐based interventions for nonfluent aphasia: A systematic review of randomized control trials
Source: Ann N Y Acad Sci. 2025 Jun 21;1549(1):92–111. doi: 10.1111/nyas.15387 (PMC12309435; doi:10.1111/nyas.15387)
Supplement: Supplementary file 1 — TABLE S1 Search strategy used for selection of articles in MEDLINE, PsycInfo, and Pubmed. [file NYAS-1549-92-s002.docx]

Supporting Table S1. Search strategy used for selection of articles in MEDLINE and PsycInfo, and Pubmed.

| Database | MEDLINE and APA PsycInfo (Ovid) | Pubmed |
| --- | --- | --- |
| Search strategy | Keyword | Query |
| Population: Non-fluent aphasia | (aphasi*).tw,kf. | aphasi* [Title/Abstract] |
|  | AND | AND |
| Concept: Music-based Interventions | (music* OR song OR melod* OR beat OR intonation OR (auditory rhythm* OR (singing or sing))).tw,kf.  AND  (therap* OR intervention* OR training OR treatment* OR rehabilitation).tw,kf. | music* [Title/Abstract] OR song*[Title/Abstract] OR melod* [Title/Abstract] OR beat [Title/Abstract] OR intonation [Title/Abstract] OR (auditory rhythm*[Title/Abstract]) OR (singing or sing [Title/Abstract])  AND  therap* [Title/Abstract] OR intervention* [Title/Abstract] OR training [Title/Abstract] OR treatment* [Title/Abstract] OR rehabilitation [Title/Abstract] |
| Limits | Publication year: 2004-Current (June 17,2024), Languages: English, Age groups: All adult (19 plus years); Publication Types: Peer-Reviewed Journal, Document Types: Journal Article”, Population Groups: Human | Publication Date: June 18, 2004-June 17,2024; Language: English; Age:19+ years; Species: Human |
