# Supplementary material for: Music‐based interventions for nonfluent aphasia: A systematic review of randomized control trials
Source: Ann N Y Acad Sci. 2025 Jun 21;1549(1):92–111. doi: 10.1111/nyas.15387 (PMC12309435; doi:10.1111/nyas.15387)
Supplement: Supplementary file 2 — TABLE S2 JBI risk of bias assessment and statistical conclusion validity questions. [file NYAS-1549-92-s001.docx]

Supporting Table S2. JBI risk of bias assessment and statistical conclusion validity questions

| **Categories** | **Questions** | **Example questions** |
| --- | --- | --- |
| **Internal validly** | | |
| **Bias related to selection and allocation** | **Q1. Was true randomization used for assignment of participants to treatment groups?** | - List of random numbers? - Computer-generated list of random numbers? - Statistician, external to the research team consulted for the randomization sequence generation? |
|  | **Q2. Was allocation to treatment groups concealed?** | - Appropriate allocation concealment procedure? - Central randomization used - Sequentially numbered, opaque and sealed envelopes? |
|  | **Q3. Were treatment groups similar at the baseline?** | - Participants from the compared groups similar with regards to the characteristics that may explain the effect even in the absence of the ‘cause’, such as age, severity of the disease, stage of the disease, co-existing conditions and so on? |
| **Bias related to administration of intervention/exposure** | **Q4. Were participants blind to treatment assignment?** | - Appropriate blinding procedure? |
|  | **Q5. Were those delivering the treatment blind to treatment assignment?** | - Any information in the article about those delivering the treatment? - Those delivering the treatment unaware of the assignments of participants to the compared groups? |
|  | **Q6. Were treatment groups treated identically other than the intervention of interest?** | - Other exposures or treatments occurring at the same time with the ‘cause’? - Plausible that the ‘effect’ may be explained by other exposures or treatments occurring at the same time with the ‘cause’? - Clear that there is no other difference between the groups in terms of treatment or care received, other than the treatment or intervention of interest? |
| **Bias related to assessment, detection and measurement of the outcome** | **Q7. Were outcome assessors blind to treatment assignment?** | - Any information in the article about outcomes assessors? - Those assessing the treatment’s effects on outcomes unaware of the assignments of participants to the compared groups? |
|  | **Q8. Were outcomes measured in the same way for treatment groups?** | - Same instrument or scale? - Same measurement timing? - Same measurement procedures and instructions? |
|  | **Q9. Were outcomes measured in a reliable way** | - The number of raters? - Training of raters? - the intra-rater and the inter-raters reliability within the study? |
| **Bias related to participant retention** | **Q10. Was follow up complete and if not, were differences between groups in terms of their follow up adequately described and analysed?** | - Description of the incomplete follow up including the number of participants and the specific reasons for loss to follow up? - Reasons for loss to follow up different? - Analysis of patterns of loss to follow up? (e.g., numbers/proportions and reasons), - Analysis of the impact of the loss to follow up on the results? |
| **Statistical Conclusion Validity** | | |
| **Statistical Aspects** | **Q11. Were participants analysed in the groups to which they were randomized?** | - ITT analysis reported? |
|  | **Q12. Was appropriate statistical analysis used?** | - Appropriate statistical power analysis? - Appropriate effect sizes used? |
|  | **Q13. Was the trial design appropriate and any deviations from the standard RCT design (individual randomization, parallel groups) accounted for in the conduct and analysis of the trial?** | - Crossover trials should only be conducted in people with a chronic, stable condition, where the intervention produces a short-term effect (i.e. relief in symptoms). - Crossover trials should ensure there is an appropriate period of washout between treatments. |
